# Age, dyslipidemia, and gamma-glutamyl transferase are associated with diabetes in obesity: a cross-sectional study

**DOI:** 10.3389/fcdhc.2026.1826118

**Published:** 2026-05-29

**Authors:** Shaomei Wang, Yongdong Su, Yanfeng Zhu, Lingyu Zhong, Xi Wang

**Affiliations:** 1 Department of Endemic and Parasitic Diseases Prevention and Control, Hechuan District Center for Disease Control and Prevention, Chongqing, China; 2 Department of Forensic Technology, Criminal Investigation Detachment, Hechuan District Public Security Bureau, Chongqing, China; 3 School of Public Health, Chengdu Medical College, Chengdu, China; 4 Department of Clinical Nutrition, Hospital of Chengdu Office of People’s Government of Tibetan Autonomous Region, Chengdu, China; 5 Department of Endocrinology, Hospital of Chengdu Office of People's Government of Tibetan Autonomous Region, Chengdu, China

**Keywords:** age, diabetes, dyslipidemia, GGT, obesity

## Abstract

**Purpose:**

Obesity can lead to various complications, particularly diabetes, which imposes a substantial health and economic burden on affected individuals. However, there remains limited research on identifying which subgroups of individuals with obesity are at higher risk of developing diabetes.

**Patients and methods:**

A convenience sampling method was used to select individuals with primary obesity from a hospital. Binary logistic regression analysis was employed to identify associated factors for the co-occurrence of diabetes and obesity.

**Results:**

The prevalence of diabetes among individuals with obesity was 20.97%. The group with both obesity and diabetes showed a higher waist-to-hip ratio (WHR) and lower muscle mass percentages in both lower limbs compared to individuals with obesity but without diabetes. Age (*OR* = 1.04; *95% CI*: 1.00–1.08), dyslipidemia (*OR* = 2.45; *95% CI:* 1.01–6.25) and gamma-glutamyl transferase (GGT) (*OR* = 1.01; *95% CI*: 1.00–1.03) were associated with diabetes comorbidity in individuals with obesity.

**Conclusion:**

Age, dyslipidemia, and GGT may help identify individuals with obesity at higher diabetes risk. Abnormal findings in these markers may warrant timely intervention to reduce diabetes incidence.

## Introduction

Obesity and diabetes have emerged as a pressing public health concern. In China, the prevalence of obesity has increased steadily over the past two decades (1), while the number of people with diabetes has reached 118 million, accounting for 22% of the global count (2). Critically, these two conditions are closely linked: individuals with obesity have a more than six-fold higher risk of developing diabetes than those without obesity (3). The combination of diabetes and obesity not only causes complications (e.g., cardiovascular and chronic kidney disease) but also drives up medical costs. By 2030, obesity−related expenses are projected to account for 21.5% of China’s total healthcare spending (4). Previous research has investigated the link between obesity and diabetes across both mechanistic and population epidemiological dimensions (5–10). However, most existing research focused on non-modifiable factors (e.g., genetics, immune mechanisms) or single obesity indicators, with insufficient attention to the comprehensive effects of modifiable and multi-dimensional factors (9, 11, 12). Addressing modifiable factors is particularly important because they offer actionable targets for clinical and public health interventions, whereas non-modifiable factors do not. Moreover, incorporating multi-dimensional measures can better capture the complex pathways linking obesity to diabetes, thereby advancing current knowledge beyond the limitations of single−indicator approaches. Furthermore, many population−based epidemiological studies may not have adequately controlled for obesity as a potential confounding factor, which could affect the accuracy of risk estimates for other exposures. This study targets individuals with obesity and analyzes the impact of modifiable factors on diabetes prevalence. By incorporating multi−dimensional modifiable risk factors, this study aims to provide a basis for the early identification of high−risk individuals with obesity and the development of stratified intervention strategies.

## Materials and methods

### Study participants

A total of 186 participants were enrolled in this study. Individuals with obesity were recruited from the multidisciplinary metabolic weight loss clinic of the Chengdu Office Hospital of the Tibet Autonomous Region between 2021 and December 2024. Inclusion criteria were: (1) aged ≥ 18 years; (2) primary obesity with a body mass index (BMI) ≥ 28 kg/m²; (3) voluntary participation in initial registration and completion of the questionnaire; (4) no psychiatric or other relevant disorders, with intact expressive and comprehension capacities. The study was approved by the Ethics Committee of Chengdu Office Hospital affiliated to the People’s Government of the Tibetan Autonomous Region (No. 2024-KY-35). Written informed consent was obtained from all participants.

### Data collection

The questionnaire, developed by Peking Union Medical College Hospital (PUMCH), included sociodemographic, behavioral, and dietary items with established content validity. Four trained registered dietitians completed 8 hours of standardized training; inter-rater reliability was excellent (ICC > 0.85, kappa > 0.80) in a pilot survey (n = 20). Data were collected via a WeChat mini-program with built-in range/logic checks, mandatory fields, and real-time error prompts. Two independent researchers cross-checked 20% of entries (concordance>98%); electronic capture precluded double-entry.

### Anthropometric and physical measurements

Height, weight, waist circumference (WC), and hip circumference (HC) were measured using calibrated instruments (SECA 213 stadiometer and SECA 803 scale; calibration checked weekly). WC was measured at the midpoint between the lowest rib and the iliac crest; HC at the maximal gluteal protrusion. Each measurement was taken twice and averaged; if the two values differed by more than 0.5 cm, a third measurement was taken. Waist‑hip ratio (WHR) was calculated as WC/HC. BMI was calculated as weight (kg)/height² (m²).

### Body composition analysis

Body composition was measured using the Inbody 230 analyzer (Biospace, Korea) following the manufacturer’s standard protocol. Key indicators included total body water (TBW), body fat mass (BFM), skeletal muscle mass (SMM), percentage body fat (PBF), basal metabolic rate (BMR), left arm muscle mass percentage (LAMMP), trunk muscle mass percentage (TMMP), right leg muscle mass percentage (RLMMP), and left leg muscle mass percentage (LLMMP).

### Biochemical analyses

Venous blood samples were collected after an overnight fast of at least 10 hours. Biochemical analyses were performed using the Beckman AU5800 analyzer (Beckman Coulter, USA). The laboratory followed the Chinese National Accreditation Service for Conformity Assessment (CNAS) quality control procedures; internal quality controls were run daily, and external quality assurance was performed quarterly. Analyzed parameters included: fasting plasma glucose (FPG), 2‑hour postprandial blood glucose (2hPBG), fasting insulin (FINS), 2‑hour postprandial insulin (2hINS), hemoglobin A1c (HbA1c), total cholesterol (TC), triglycerides (TG), high‑density lipoprotein cholesterol (HDL‑C), low‑density lipoprotein cholesterol (LDL‑C), very low‑density lipoprotein cholesterol (VLDL‑C), aspartate aminotransferase (AST), alanine aminotransferase (ALT), urea, creatinine (Cr), estimated glomerular filtration rate (eGFR), and uric acid (UA).

### Sample size calculation

The sample size was estimated using the method proposed by Hsieh et al. for logistic regression. Based on previous literature, the odds ratio (*OR*) for elevated GGT and diabetes was approximately 2.29 (13). We set a two-sided significance level (*α*) of 0.05, a power (1-*β*) of 0.80, a diabetes prevalence of 9.6% among individuals with obesity (14), and a coefficient of determination (ρ²) of 0.10 to account for the effect of other covariates on GGT. The calculated minimum required sample size was approximately 146 cases. Finally, a total of 186 participants were enrolled in this study, meeting the minimum sample size requirement.

### Diagnostic criteria

#### Obesity

According to the expert consensus on obesity prevention and treatment for Chinese residents (15), overweight was defined as a BMI of 24.0 to 27.9 kg/m²; obesity was defined as a BMI of ≥ 28.0 kg/m².

#### Diabetes

Based on the guidelines for the prevention and treatment of type 2 diabetes in China (2020 Edition) (16), the diagnostic criteria were as follows. In individuals with typical diabetic symptoms (polydipsia, polyuria, polyphagia, and unexplained weight loss), a single measurement meeting any of the following was diagnostic: random blood glucose ≥11.1 mmol/L, fasting plasma glucose (FPG) ≥7.0 mmol/L, 2−hour postprandial glucose ≥11.1 mmol/L during an oral glucose tolerance test (OGTT), or HbA1c ≥6.5%. For asymptomatic individuals, diagnosis required confirmation by a second abnormal test on a separate day (using the same criterion).

#### Dyslipidemia

According to the 2016 revised Chinese guidelines for the management of dyslipidemia in adults (17), the diagnosis was defined by the presence of any of the following: TC ≥6.2 mmol/L, LDL-C ≥4.1 mmol/L, HDL-C ≤1.0 mmol/L, or TG ≥2.3 mmol/L.

### Statistical analysis

Data processing and statistical analyses were performed using R (Version 4.2.3). Normality of continuous data was assessed by Shapiro–Wilk test; all continuous variables met this assumption and were compared between groups using the independent samples t-test (mean ± SD). Categorical data were presented as n (%) and compared by chi-square test (or Fisher’s exact test). Missing data accounted for 1.6% of all values and were handled using multiple imputation. Binary logistic regression was used to identify factors associated with diabetes–obesity co−occurrence. Variables were selected based on clinical relevance and univariate screening (*p<* 0.10). Multicollinearity was assessed via variance inflation factors (VIF); all VIF values ranged from 1.05 to 1.56 (all<5), indicating no significant collinearity. Model fit was evaluated by Hosmer–Lemeshow test (*p* > 0.05 indicating good fit). Effect sizes were reported as adjusted odds ratios (*OR*) with 95% confidence intervals (CI). Significance level was *α* = 0.05 (two−tailed).

## Results

### Description of characteristics of participants

A total of 186 individuals with obesity were included in the final analysis, of whom 75 were male (40.32%) and 111 were female (59.68%). The prevalence of diabetes among the individuals with obesity was 20.97% (39/186). Diabetes prevalence was significantly higher in males, married individuals, those with dyslipidemia, and older participants. No other variables differed significantly between groups (**Table 1**).

**Table 1:** General demographic characteristics of participants n(%).

Items		Without diabetes	With diabetes	χ^2^	*P*-value
Gender				6.19	0.01
	Male	52(35.37)	23(58.97)		
	Female	95(64.63)	16(41.03)		
Ethnicity				0.05	0.83
	Han	58(39.46)	14(35.90)		
	Tibetan	89(60.54)	25(64.10)		
Marital status		97(65.99)	34(87.18)	5.67	0.02
History of family obesity		76(51.70)	23(58.97)	0.40	0.53
Hyperuricemia		52(35.37)	13(33.33)	0.002	0.96
Dyslipidemia		67(45.58)	29(74.36)	9.10	<0.01
Obstructive sleep apnea syndrome		29(19.73)	9(23.08)	0.06	0.81

### Description of biochemical indicators of participants

Biochemical parameters were compared between the groups with and without diabetes. Levels of FPG, 2hPBG, HbA1c, GGT, and eGFR were significantly higher in the group with diabetes than in the group without diabetes, whereas Cr levels were markedly lower (*p* < 0.05). Despite the intergroup differences in eGFR and Cr, both indices remained within the clinical reference ranges and therefore had no practical clinical significance. No statistically significant differences were observed between the two groups in lipid profiles or other liver function parameters (**Table 2**).

**Table 2:** Comparison of biochemical parameters between groups with and without diabetes ( $\bar{X}\pm$ S).

Items	Total	Without diabetes	With diabetes	*t*	*P*-value
FPG (mmol/L)	5.62 ± 1.02	5.34 ± 0.59	6.69 ± 1.50	-5.55	<0.01
2hPBG (mmol/L)	8.51 ± 3.52	7.41 ± 2.39	12.64 ± 4.01	-7.78	<0.01
HbA1c (%)	6.67 ± 1.56	6.43 ± 1.35	7.57 ± 1.94	-3.44	<0.01
ALT (U/L)	42.68 ± 30.54	42.90 ± 31.56	41.85 ± 26.69	0.21	0.83
AST (U/L)	25.61 ± 12.30	25.73 ± 12.31	25.15 ± 12.38	0.26	0.80
GGT (U/L)	50.11 ± 34.55	46.13 ± 32.07	65.08 ± 39.60	-2.76	<0.01
Cr (μmol/L)	65.75 ± 19.32	67.29 ± 20.40	59.97 ± 13.23	2.70	<0.01
UA (μmol/L)	403.99 ± 104.58	405.82 ± 109.06	397.13 ± 86.51	0.53	0.60
eGFR (ml/min/1.73m^2^)	112.72 ± 22.54	110.09 ± 19.87	122.61 ± 28.78	-2.56	0.01
TC (mmol/L)	4.46 ± 0.92	4.43 ± 0.93	4.59 ± 0.85	-1.05	0.30
TG (mmol/L)	1.57 ± 0.87	1.52 ± 0.88	1.72 ± 0.84	-1.30	0.20
HDL-C (mmol/L)	1.20 ± 0.23	1.21 ± 0.23	1.16 ± 0.24	1.20	0.23
LDL-C (mmol/L)	2.86 ± 0.74	2.84 ± 0.73	2.92 ± 0.77	-0.55	0.59

### Comparison of body composition between groups with and without diabetes

Body composition parameters were compared between individuals with diabetes and those without diabetes. The group with diabetes had a significantly higher WHR and lower muscle mass percentages in both lower limbs compared with the group without diabetes (*p* < 0.05). No statistically significant differences were detected in other body composition parameters between the two groups (**Table 3**).

**Table 3:** Comparison of body composition parameters between groups with and without diabetes ( $\bar{X}\pm$ S).

Variables	Total	Without diabetes	With diabetes	*t*	*P*-value
Height (cm)	165.03 ± 8.49	164.66 ± 8.55	166.44 ± 8.25	-1.19	0.24
Weight (kg)	89.01 ± 14.89	88.40 ± 15.11	91.29 ± 14.00	-1.13	0.26
BMI (kg/m^2^)	32.67 ± 4.04	32.58 ± 4.18	33.03 ± 3.47	-0.69	0.49
WHR	1.00 ± 0.07	0.99 ± 0.07	1.02 ± 0.07	-2.71	0.01
BMR [KJ/(m^2^.h)]	1507.73 ± 222.14	1497.75 ± 226.65	1545.36 ± 202.55	-1.27	0.21
TBW (%)	38.65 ± 7.56	38.31 ± 7.72	39.95 ± 6.86	-1.29	0.20
Protein (kg)	10.39 ± 2.07	10.30 ± 2.11	10.72 ± 1.89	-1.21	0.23
Minerals (kg)	3.62 ± 0.68	3.59 ± 0.68	3.74 ± 0.64	-1.23	0.22
BFM (kg)	36.34 ± 8.80	36.19 ± 9.05	36.88 ± 7.85	-0.47	0.64
FFM (kg)	52.67 ± 10.28	52.21 ± 10.49	54.41 ± 9.37	-1.27	0.21
SMM (kg)	29.35 ± 6.25	29.08 ± 6.38	30.36 ± 5.70	-1.21	0.23
PBF (%)	40.75 ± 6.47	40.87 ± 6.63	40.32 ± 5.90	0.50	0.62
PRAMM (%)	109.99 ± 10.80	110.04 ± 10.52	109.80 ± 11.93	0.11	0.91
LAMMP (%)	109.02 ± 10.50	109.01 ± 10.17	109.09 ± 11.79	-0.04	0.97
TMMP (%)	103.99 ± 5.32	104.11 ± 5.25	103.55 ± 5.61	0.56	0.58
RLMMP (%)	97.33 ± 7.22	98.01 ± 7.18	94.73 ± 6.87	2.63	0.01
LLMMP (%)	96.38 ± 6.82	96.93 ± 6.77	94.34 ± 6.68	2.15	0.04

### Diabetes risk factors in individuals with obesity

Binary logistic regression analysis was conducted with the presence of diabetes as the dependent variable in individuals with obesity, after adjusting for potential confounding factors including sex, ethnicity, family history of obesity and dietary habits. The results demonstrated that age (*OR* = 1.04, *95% CI*: 1.00–1.08), dyslipidemia (*OR* = 2.45, *95% CI*: 1.01–6.25), and GGT (*OR* = 1.01, *95% CI*: 1.00–1.03) significantly increased the risk of diabetes comorbidity in individuals with obesity. Specifically, each one-year increase in age corresponded to an approximately 4% increase in the odds of diabetes; the presence of dyslipidemia was associated with 2.45−fold higher odds compared with absence; and each 1 U/L elevation in GGT corresponded to a 1% increase in the odds of diabetes (**Table 4**).

**Table 4:** Binary logistic regression analysis of risk factors for diabetes in individuals with obesity.

Variable	*OR*	95% *CI*	*P*-value
Age	1.04	1.00-1.08	0.03
Gender	0.75	0.31-1.81	0.52
Ethnicity	0.93	0.40-2.20	0.87
Marital status	1.81	0.53-6.91	0.36
Family history of obesity	1.52	0.69-3.43	0.31
Drinking status	1.01	0.44-2.33	0.97
Oil-salt preference	0.82	0.41-1.68	0.59
Snack habit	0.83	0.49-1.39	0.49
BMI	1.04	0.94-1.12	0.43
Dyslipidemia	2.45	1.01-6.25	0.05
GGT	1.01	1.00-1.03	0.02

### Correlation analysis of blood lipids and diabetes-related biomarkers

**Figure 1:**
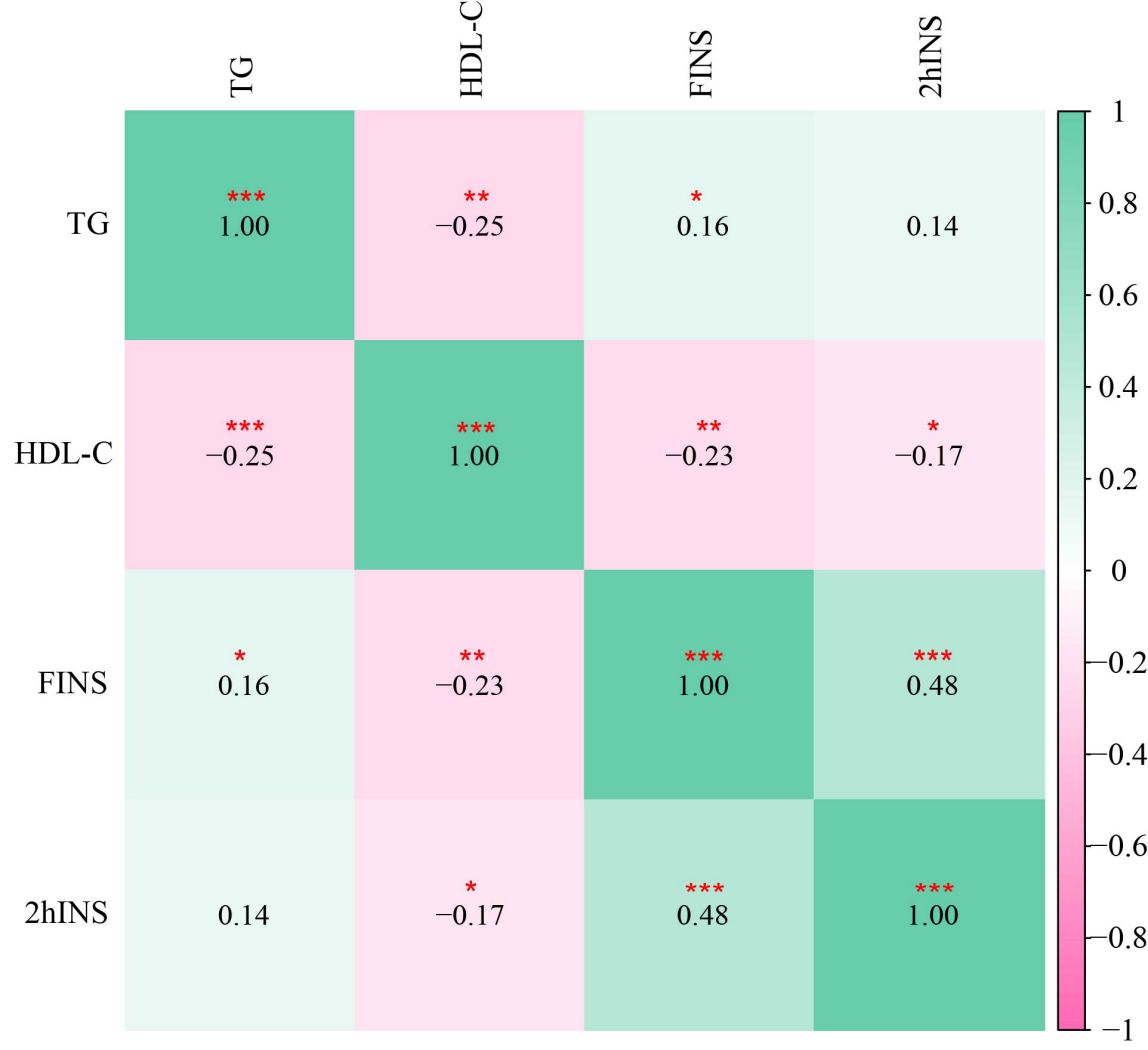
Correlation between blood lipids and diabetes-related parameters.

Correlation analyses were performed between blood lipid indicators and diabetes-related biomarkers. TG was positively correlated with FINS, while HDL-C was negatively correlated with both FINS and 2hINS (**Figure 1**).

## Discussion

In our study, the WHR was significantly higher in the group with diabetes, consistent with prior evidence linking central obesity to increased diabetes risk (18), diabetes onset, and elevated glycemic levels (19). Therefore, WC should be emphasized alongside BMI in clinical management to reduce visceral fat and diabetes risk. Beyond central obesity, individuals with diabetes also had significantly lower muscle mass percentages in both lower limbs, consistent with reports that diabetes preferentially affects leg muscles and leads to faster muscle loss and higher sarcopenia prevalence (20–22). This suggests that weight management in individuals with obesity should prioritize fat reduction while preserving muscle mass.

Beyond body composition, our regression analysis identified three factors independently associated with diabetes in individuals with obesity: age, dyslipidemia, and GGT. A nationwide cohort study showed that diabetes incidence increases with age, which aligns with known declines in insulin sensitivity and *β*-cell function (23–25). In addition to age, individuals with dyslipidemia had a higher risk of diabetes. TG was positively correlated with FINS, while HDL-C was negatively correlated with both FINS and 2hINS, aligning with previous reports linking dyslipidemia to insulin resistance (26–32). Additionally, GGT was associated with diabetes in our study, consistent with previous reports linking elevated GGT to impaired fasting glucose, insulin resistance, and oxidative stress (33–35). While age is non-modifiable, dyslipidemia and GGT are routine, low-cost biomarkers that can be acted upon. The GGT finding is particularly notable: in obese individuals without classic risk factors, a marginal GGT elevation may serve as an early warning of oxidative stress and insulin resistance, prompting earlier screening. Adding GGT to age and lipid assessment offers a practical risk-stratification tool, especially in resource-limited settings.

## Limitations

Several limitations should be acknowledged. First, the cross−sectional design precludes causal inference; we therefore interpret all associations as correlates rather than causes and we used binary logistic regression to adjust for potential confounders. Second, convenience sampling from a single hospital clinic may limit generalizability. To mitigate this, we applied strict inclusion and exclusion criteria and provided detailed participant characteristics to facilitate comparison with other populations. Third, we could not collect data on medication use (e.g., antidiabetic, lipid−lowering drugs), quantitative physical activity, or detailed dietary intake, which are potential confounders. Nonetheless, we adjusted for closely related lifestyle factors—drinking status, oil−salt preference, snack habits—and BMI, which may partially reduce residual confounding. Fourth, the sample size is modest (n=186, with only 39 diabetes events), which raises concerns regarding statistical power and the stability of the regression model. To address this, we prespecified all covariates based on clinical relevance rather than data−driven selection. Nevertheless, given the limited events−per−variable ratio, our findings should be interpreted as exploratory and hypothesis−generating, and future prospective, multi−center studies with larger samples and comprehensive covariate assessment are needed to validate and generalize our findings.

## Conclusion

In this study of individuals with obesity, those with comorbid diabetes had poorer glycemic control, a higher WHR, and significantly lower muscle mass percentages in both lower limbs compared to those without diabetes. Binary logistic regression identified three independent factors associated with diabetes in this population: age, dyslipidemia, and GGT. Additionally, TG was positively correlated with FINS, while HDL-C was negatively correlated with FINS and 2hINS. These findings highlight that, beyond general obesity measures, simple clinical indicators—age, lipid profile, and GGT—can help identify individuals with obesity at higher diabetes risk. Notably, the muscle loss in the lower limbs observed in the diabetic subgroup suggests that preserving muscle mass should be integrated into weight management strategies. Taken together, these readily available markers offer a practical, low-cost approach for risk stratification in clinical and community settings, particularly where advanced testing is limited.

## Data Availability

The raw data supporting the conclusions of this article will be made available by the authors, without undue reservation.
